# A new SO_2_ probe ZSO targeting VDBP inhibits high glucose induced endothelial cell senescence and calcification

**DOI:** 10.3389/fphys.2025.1719853

**Published:** 2026-01-05

**Authors:** Yangyang Zhang, Xiaomeng Yan, Xinyu Dong, Congyao Zhao, Xiaohui Chi, Baoxiang Zhao, Junying Miao, Zhaomin Lin

**Affiliations:** 1 Shandong Provincial Key Laboratory of Development and Regeneration, School of Life Science, Shandong University, Qingdao, China; 2 Institute of Organic Chemistry, School of Chemistry and Chemical Engineering, Shandong University, Jinan, China; 3 Institute of Medical Science, The Second Hospital of Shandong University, Jinan, China

**Keywords:** calcification, db/db mice, senescence, vascular endothelial cells, vitamin D binding protein

## Abstract

**Introduction:**

Vitamin D binding protein (VDBP) serves as a key biomarker for vascular injury, offering important applications in both diagnosis and prognosis. However, its precise functional role remains incompletely understood.

**Methods:**

We investigated the compound ZSO as a VDBP-interacting compound conferring potent anti-senescence effects. Given that endothelial senescence and calcification drive vascular aging, processes accelerated by high glucose. We assessed the impact of ZSO *in vitro* using high glucose-induced vascular endothelial cell models of senescence and calcification and *in vivo* using a db/db mouse model of vascular aging and calcification.

**Results:**

ZSO treatment markedly alleviated high glucose-induced endothelial cell senescence and calcification *in vitro* and suppressed vascular aging and calcification in db/db mice *in vivo*. Mechanistic investigations revealed that high glucose-induced vascular endothelial senescence and calcification were accompanied by upregulated VDBP protein levels. The compound ZSO bound to pathologically elevated VDBP in senescent endothelial cells and db/db mice, triggering its ubiquitin-mediated proteasomal degradation without altering transcriptional regulation.

**Discussion:**

This study identifies ZSO as a novel small molecule that inhibits endothelial senescence and calcification, and establishes its role in directly targeting VDBP to promote degradation. Furthermore, our findings underscore the critical role of VDBP in vascular aging and calcification and suggest its potential utility as a biomarker for diabetic vascular complications.

## 1 Introduction

Vascular diseases have become a leading cause of long-term disability and mortality in aging populations, highlighting the importance of maintaining vascular health in an aging society (Morrish et al., 2001; Lakatta and Levy, 2003). Vascular endothelial cells, which line the inner surface of blood vessels, are exposed to a dynamic and complex microenvironment (Bloom et al., 2023). Constant exposure to high glucose (HG) accelerates endothelial cell senescence (Hobson and Denekamp, 1984; Woywodt et al., 2002; Li et al., 2022). Vascular calcification, marked by the deposition of calcium phosphate complexes in blood vessels, results from disturbances in mineral balance (Lee et al., 2020; Villa-Bellosta, 2021; Yuan et al., 2021). Currently, most research focuses on the transdifferentiation of vascular smooth muscle cells (VSMCs) into osteoblast- or chondrocyte-like cells (Giachelli, 2004; Leopold, 2015; Lee et al., 2020; Yuan et al., 2021; Gui et al., 2024), which then migrate to the intima and form calcified foci (Thompson and Towler, 2012; Chavkin et al., 2015). Recent studies have also proposed that endothelial cells, under certain conditions, may contribute to vascular calcification (Albiero et al., 2014; Cianciolo et al., 2016; Hong et al., 2018; Yuan et al., 2021). However, the role of endothelial cells in vascular calcification still requires further study.

Vitamin D binding protein (VDBP), also known as Gc-globulin, is a plasma glycoprotein that primarily serves to transport vitamin D and its metabolites (Braun et al., 1993; Song et al., 1999; DiMartino et al., 2007; Bikle and Schwartz, 2019). Previous studies have demonstrated that VDBP level is altered in various diseases, reflecting the severity of these conditions (Lisowska-Myjak et al., 2020; Agliardi et al., 2023). Notably, elevated VDBP level has been associated with vascular injury, such as in fresh thrombotic plaques and ST-segment elevation myocardial infarction (STEMI) (Stakisaitis et al., 2016). Additionally, in chronic kidney disease, VDBP level correlates with the severity of diabetic nephropathy (Kalousova et al., 2015). Given its role in modulating vitamin D bioavailability and calcium-phosphorus balance (Martínez-Sena et al., 2020), VDBP is hypothesized to be a potential influencer of vascular calcification.

Given the potential involvement of VDBP in vascular calcification and related pathologies, there is a growing need for targeted molecular tools to investigate its functional roles and facilitate therapeutic intervention. In our previous work, we synthesized and characterized a novel ratiometric fluorescent probe 4-methylbenzo [4,5] imidazo [1,2-α] pyridine-3-carbaldehyde (ZSO) for the rapid, sensitive, and selective detection of endogenous sulfur dioxide (SO_2_) in live cells (Cao et al., 2016). Importantly, this study demonstrated that ZSO specifically binds to VDBP and identified ZSO as a novel inhibitor of vascular endothelial cell senescence and calcification, offering new insights for developing anti-aging and anti-calcification therapeutics targeting VDBP. Therefore, this study aims to elucidate the specific role and mechanism by which ZSO, through its binding to VDBP, inhibits high glucose-induced endothelial cell senescence and calcification, thereby providing a potential therapeutic strategy for treating vascular senescence and calcification pathologies.

## 2 Materials and methods

### 2.1 Chemicals

ZSO was synthesized according to established protocols and dissolved in DMSO to prepare a 0.1 M stock solution (Cao et al., 2016).

### 2.2 Antibodies

p21 (10355-1-AP) and BMP2 (66383-1-Ig) antibodies were purchased from Proteintech. RUNX2 (12556S) antibodies were obtained from Cell Signaling Technology. VDBP antibodies were acquired from Zhengneng Biotechnology (R381684) and Santa Cruz Biotechnology (sc-365441). GAPDH (A19056) antibody was purchased from ABclonal, and CD31 (3528S) antibody was from Cell Signaling Technology. Ubiquitin (ab137031) antibodies were sourced from Abcam.

### 2.3 Cell culture

Human umbilical vein endothelial cells (HUVECs) were obtained from Fuheng Biotechnology (FH1122) and cultured in gelatin-coated dishes using M199 medium supplemented with 15% fetal bovine serum (FBS) (Zhao X. et al., 2020; Mu et al., 2024). Cells were cultured in an incubator at 37 °C and 5% CO_2_.

### 2.4 Sulforhodamine B assay

HUVEC cells were incubated in 96-well plates for 24 h and then incubated with ZSO (0, 0.1, 0.5, 1, 5 and 10 μM) for 24 h under high glucose conditions. After terminating cell culture, the medium was removed and the cells were fixed with 100 µL 10% trichloroacetic acid (TCA) for 1 h at 4 °C. The wells were gently rinsed five times with deionized water. 50 μL of SRB dye solution was added to each well and incubated for 10 min at room temperature. Wash unbound dye thoroughly using 1% glacial acetic acid. Solubilize bound SRB by adding 100 µL 10 mM Tris base/buffer. Measure the optical density at 540 nm.

### 2.5 Western blot

Western blotting followed published protocols (Kong et al., 2019; Cui et al., 2022). HUVECs were seeded into plates and treated under specific conditions after reaching confluence. Cells were washed three times with 1×PBS and lysed using Western & IP lysis buffer containing 1% protease inhibitor cocktail. After complete lysis, the cell lysate was transferred to a 1.5 mL EP tube. Lysates were centrifuged at 12,000 rpm (4 °C, 15 min), and supernatants were collected. Protein content was measured by use of the BCA Protein Assay Kit (Beyotime, P0011). Samples were mixed with loading buffer, boiled at 100 °C for 5 min, and separated on 10% SDS-PAGE gels (150 V, 1 h). Proteins were transferred to PVDF membranes (160 mA, 2.5 h), blocked with 5% skim milk (2 h, RT) and incubated with primary antibodies (4 °C, overnight). After washing with TBST, membranes were incubated with secondary antibodies for 1 h at room temperature. Protein bands were visualized using an imaging system.

### 2.6 SA-β-Gal staining

The senescence-associated β-galactosidase (SA-β-Gal) staining kit (Beyotime, C0602) was used for SA-β-Gal staining of cells and frozen sections.

### 2.7 Calcium ion level detection

HUVECs were seeded in culture dishes for confocal laser scanning microscopy. After cells grew to the appropriate density endothelial cells were treated under calcifying conditions with/without ZSO for 4 days. Cells were gently washed with RR buffer after the culture medium had been discarded. Then, 500 μL of calcium ion dye solution was added. Cells were incubated at 37 °C under dark conditions for 30 min. The calcium ion dye solution was aspirated, and the cells were gently washed three times with RR buffer. Then, 500 μL of RR buffer was added to each well and incubated in the dark for 30 min for deesterification. Subsequently, the calcium ion level in endothelial cells was detected using a confocal laser scanning microscope (488 nm excitation).

### 2.8 Molecular docking

Molecular docking between ZSO and VDBP (PDB: 1J7E) was performed in AutoDock-GPU (Zhao Y. et al., 2020). The receptor was prepared by removing water and adding polar hydrogens. Docking employed the Lamarckian Genetic Algorithm with the binding pocket defined by the co-crystal ligand position. We executed 100 independent runs per ligand and selected the conformation with the optimal docking score as the most probable binding complex. Interaction analysis used Discovery Studio Visualizer and PyMOL for 3D rendering.

### 2.9 Biacore surface plasmon resonance (SPR) experiment

VDBP protein (1 mg/mL) was dissolved in ddH_2_O and pre-coupled to a sensor chip using sodium acetate buffers. The protein was manually coupled to the chip. After protein coupling, the same tube of protein was injected repeatedly to observe the stability of the chip. ZSO was diluted with PBS containing 0.1% DMSO in a two-fold gradient, setting five concentrations: 0.016, 0.08, 0.4, 2, and 10 μM, and injected. Binding affinity was analyzed using Biacore evaluation software.

### 2.10 RT-qPCR

Total RNA was extracted using the total RNA extraction kit (BioFlux, BSC52M1). Reverse transcription of RNA into cDNA according to the instructions of HiScript RAll-in-one RT SuperMix Perfect for q-PCR (Vazyme, R333-01). The primers used are listed in Table 1. Cycling conditions: 50 °C for 15 min, 85 °C for 5 s, then 40 cycles of 95 °C (10 s) followed by 60 °C (30 s). PCR system was prepared according to the instructions of UitraSYBR Mixture (CWBio, CW0957S). RT-qPCR was performed using Bio-Rad PCR instrument.

**TABLE 1:** The sequences of primers used for RT-qPCR.

Gene	Primer type	Sequence (5′-3′)
VDBP	Forward primer	ATCTGGGAAAGGAGGACTTCACATC
VDBP	Reverse primer	CAGCAGGCTTCGGTCAAGGAG
β-actin	Forward primer	CCTGGCACCCAGCACAAT
β-actin	Reverse primer	GCCGATCCACACGGAGTACT

### 2.11 Immunoprecipitation

HUVECs were plated in 6-well plates and exposed to drugs when they reached the appropriate density. Proteins were extracted using the same method as in the Western blot experiment, and protein concentration was measured. Cell lysates (500 μg protein) were incubated with Protein A/G Magnetic Beads (MCE, HY-K0202) and VDBP antibody (Santa Cruz, sc-365441) overnight. Beads were washed, and Western blot was used for detection, first incubated with ubiquitination antibody (Abcam, ab137031), developed, and then incubated with VDBP antibody (Santa Cruz, sc-365441) and developed.

### 2.12 Animal experiments

We purchased male db/m (10-week-old) mice and male db/db mice (10-week-old). They were fed normally for 2 weeks to adapt to the environment, and then intraperitoneal injection of drugs was started. The mice were housed at a temperature of 20 °C ± 2 °C, a humidity of 55% ± 5%, and a 12 h light/dark cycle, and fed an ordinary diet. db/m mice (n = 8) received the vehicle (2% DMSO +40% PEG300 + 5% Tween 80 + 53% PBS). db/db mice were divided into three groups (n = 8 mice/group): the vehicle group, ZSO (0.2 mg/kg), and ZSO (1 mg/kg) groups, injected intraperitoneally for 8 weeks. The mean initial body weights for each group were as follows: db/m group, 25.62 ± 1.04 g; db/db + vehicle group, 50.64 ± 1.75 g; db/db + ZSO (0.2 mg/kg) group, 49.62 ± 1.13 g; db/db + ZSO (1 mg/kg) group, 49.96 ± 1.59 g. Body weight and water intake were monitored weekly. Mice were fasted overnight before euthanasia, which was performed via intraperitoneal injection of pentobarbital sodium at a dose of 150 mg/kg, and their fasting blood glucose was measured. The mice were anesthetized and blood was collected via retro-orbital bleed/from the retro-orbital plexus. After centrifugation, the serum was collected and stored at −80 °C. Tissues (Heart, liver, spleen, lung, kidney, thoracic aorta) were harvested for histology. All procedures complied with ARRIVE guidelines and were approved by the Shandong University Ethics Committee.

### 2.13 Von Kossa staining

Paraffin-embedded aortic sections were deparaffinized, rehydrated, and stained using the Calcium salt staining kit (Von Kossa method) (Solarbio, G3282). After sealing, the staining of the vessels was observed using the bright-field of a research-grade inverted fluorescence microscope.

### 2.14 ELISA

Serum VDBP level was quantified using a DBP ELISA Kit (enzyme-linked immunosorbent assay) (Wuhan Cloud-Clone, SEB810Mu).

### 2.15 Enface staining

Enface Staining was performed according to previously described protocols (Li et al., 2013). Take the thoracic aorta of mice, remove the fat thoroughly, make a longitudinal incision, and fix it at room temperature with 4% paraformaldehyde for 30 min. Wash twice with 1×PBS, permeate with 0.2% Triton X-100 for 5 min, and then wash twice with 1×PBS. The blood vessels were blocked in 10% goat serum (45 min) followed by primary antibody incubation (4 °C, overnight). Wash twice with 1×PBST and then incubate with the corresponding secondary antibody in a dark room at 37 °C for 1 h. After washing twice with 1×PBST, DAPI was added for nuclear staining. The blood vessels were attached with the anti-quenching agent, and the endothelial side was facing the lid. Blood vessels were observed using a laser confocal scanning microscope.

### 2.16 Hematoxylin and eosin (H&E) staining

Paraffin sections of mouse blood vessels, heart, liver, spleen, lung, and kidney were stained using Solarbio’s Modified Hematoxylin and Eosin (HE) Staining Kit (G1121). Following routine procedures including dewaxing and rehydration, staining, dehydration and clearing, the sections were mounted. Whole-slide scanning was then performed using a slide scanner to observe and examine the structural features of the mouse blood vessels, heart, liver, spleen, lung, and kidney.

### 2.17 Immunohistochemistry

Tissue sections were dewaxed in xylene, rehydrated through a descending ethanol series, and rinsed in distilled water. Mouse aortic sections underwent microwave-based antigen retrieval in citrate buffer (pH 6.0). After blocking with 10% goat serum and TBS rinse, primary antibodies were incubated overnight at 4 °C. Slides were PBST-washed, incubated with PBS-diluted secondary antibodies (1:200) for 1 h, then developed with DAB substrate. Reactions were water-rinsed upon positive staining observation. Nuclei were hematoxylin-counterstained, dehydrated through an ascending ethanol series, cleared in xylene, and mounted in neutral balsam. Slides were digitally scanned to document immunostaining patterns.

### 2.18 Statistical analysis

The results of at least three independent replicated experiments were taken and the differences between the groups were analysed using Graphpad Prism software, One-way ANOVA followed by Tukey’s post-hoc test. The results were expressed as mean ± SEM. *p < 0.05; **p < 0.01; ***p < 0.001; ns, p > 0.05, no significant difference.

## 3 Results

### 3.1 ZSO binds to vitamin D binding protein

**FIGURE 1:**
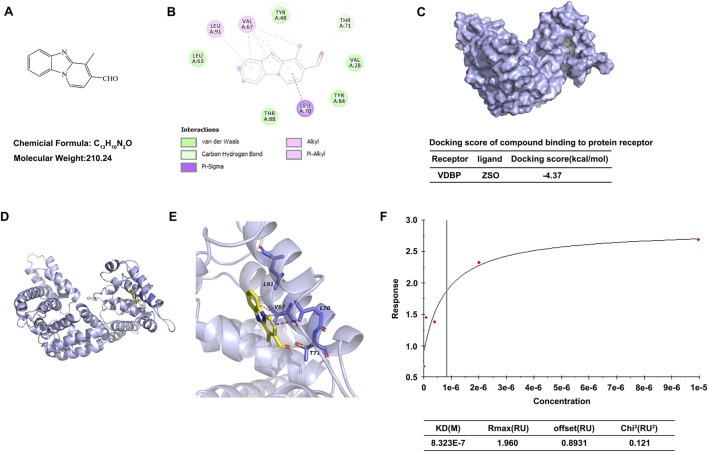
ZSO binds to Vitamin D Binding Protein. **(A)** Molecular structure of ZSO. **(B)** Two-dimensional diagram depicting the interaction between ZSO and VDBP. Light green dashed lines represent conventional hydrogen bonds. Pink and purple dashed lines denote hydrophobic interactions. Darker green residues indicate residues interacting with ZSO via van der Waals forces. **(C–E)** Three-dimensional views of the ZSO-VDBP interaction interface. The VDBP backbone is displayed as a light blue surface with cartoon representation. ZSO is shown as a yellow stick model. Residues lining the binding pocket are rendered in slate blue sticks. Light green dashed lines represent conventional hydrogen bonds; pink and purple dashed lines indicate hydrophobic interactions. **(F)** Validation of the binding between ZSO and VDBP using Biacore analysis. VDBP protein was immobilized on the chip, and the response values for ZSO binding at different concentrations were measured.

Previous studies have established VDBP as a validated biomarker of vascular injury (Huang et al., 2011; Stakisaitis et al., 2016; Lisowska-Myjak et al., 2020). Subsequently, a compound library was screened, leading to the identification of ZSO as a VDBP-binding molecule. The structural formula of ZSO is presented in Figure 1A. Molecular docking simulations were employed to predict the binding potential between ZSO and VDBP. The resultant docking score of −4.37 indicates favorable binding affinity. As shown in Figure 1B, ZSO forms a hydrogen bond with Thr71 in VDBP and interacts with Val67, Leu91, and Leu70 in VDBP through hydrophobic interactions, including alkyl, π-alkyl, and π-σ hydrophobic interactions. ZSO also interacts with Leu63, Tyr48, Val28, Tyr84, and Thr88 in VDBP through van der Waals forces (Figures 1B–E). Subsequently, the interaction was further validated using surface plasmon resonance (SPR) assays on a Biacore platform. The experimental results confirmed that ZSO binds specifically to the VDBP protein. The dissociation constant (KD) value reached the nanomolar level, and Chi^2^ (RU^2^) < 1/10 Rmax, indicating that the experimental data is reliable (Figure 1F). Collectively, these results demonstrate that ZSO binds directly to VDBP.

### 3.2 ZSO exhibits minimal toxicity *in vitro* and *in vivo* during vascular assessment

**FIGURE 2:**
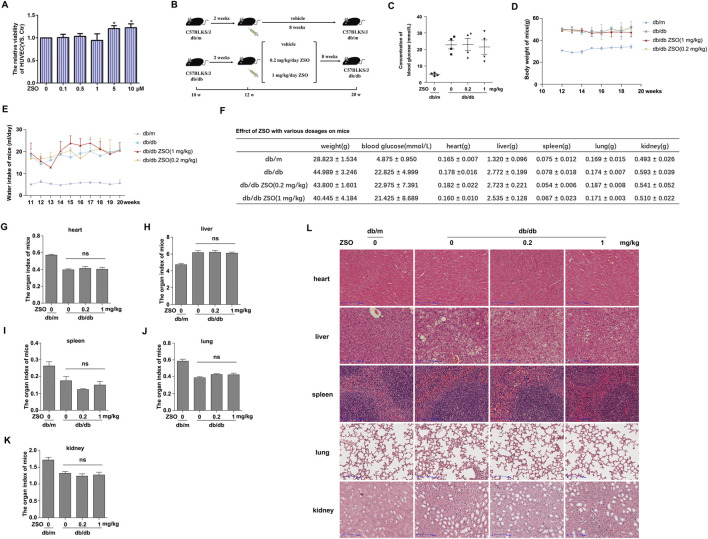
ZSO exhibits minimal toxicity *in vitro* and *in vivo* during vascular assessment. **(A)** Under high-glucose conditions (30 mM), HUVECs were treated with different concentrations of ZSO. After 24 h, cell viability was assessed using the sulforhodamine B (SRB) assay. *p < 0.05, n = 3. **(B)** Experimental design diagram outlining the mouse treatment protocol. **(C)** Fasting blood glucose level was measured after overnight fasting prior to euthanasia. **(D)** Body weight of mice. Body weight was measured and recorded weekly, followed by statistical analysis. **(E)** Water intake of mice. Water consumption was recorded weekly and analyzed statistically. **(F)** Body weight, fasting blood glucose, and organ weights at the time of sacrifice. Data are presented as mean ± standard deviation (SD). **(G–K)** Organ indices of mice, including the heart, liver, spleen, lungs, and kidneys, respectively. Organ index = organ weight/body weight × 100%. ns, p > 0.05, n = 4. **(L)** The hearts, livers, spleen, lungs and kidneys of mice were taken for paraffin sections, HE staining was performed, and then scanned using a digital section scanner to observe the structure of each organ of the mice. Scar bar = 100 μm.

The toxicity profile of ZSO was evaluated both *in vitro* and *in vivo*. We used an *in vitro* model of high glucose-induced senescence in vascular endothelial cells and an *in vivo* model employing db/db mice (a well-established model system reported to develop hyperglycemia-induced vascular injury and calcification) (Boström et al., 2011; Ren et al., 2022). The results demonstrated that ZSO had no significant cytotoxicity on endothelial cells. Furthermore, treatment with 5 μM and 10 µM ZSO significantly promoted endothelial cell proliferation (Figure 2A). For the *in vivo* study, db/db mice were divided into three groups and administered intraperitoneal injections every other day for a total of 8 weeks. Body weight and water intake were recorded weekly. Following the 8-week treatment period, mice were euthanized, and major organs (Heart, liver, spleen, lungs, kidneys) and thoracic aortic tissues were harvested (Figures 2B–L). The night prior to euthanasia, mice were fasted. Fasting blood glucose levels were measured via tail vein blood sampling, and the results showed that ZSO administration did not significantly alter blood glucose levels (Figure 2C). Throughout the study, no significant differences in body weight or water intake were observed among the groups (Figures 2D,E). Following organ collection, the heart, liver, spleen, lungs, and kidneys were weighed. Organ-to-body weight ratios (Organ coefficients) were calculated. The results showed that ZSO injection did not induce significant alterations in organ coefficients (Figures 2F–K). H&E staining was performed on paraffin sections of the heart, liver, spleen, lung, and kidney in mice (Figure 2L). Compared with vehicle-injected db/db controls, mice treated with different doses of ZSO showed no significant alterations in organ-to-body weight ratios, tissue architecture, or pathological lesions in the heart, liver, spleen, lungs, or kidneys. Collectively, these results demonstrate that intraperitoneal administration of ZSO induced no significant organ toxicity in mice.

### 3.3 ZSO inhibits high glucose-induced senescence in vascular endothelial cells and thoracic aortic senescence in db/db mice

**FIGURE 3:**
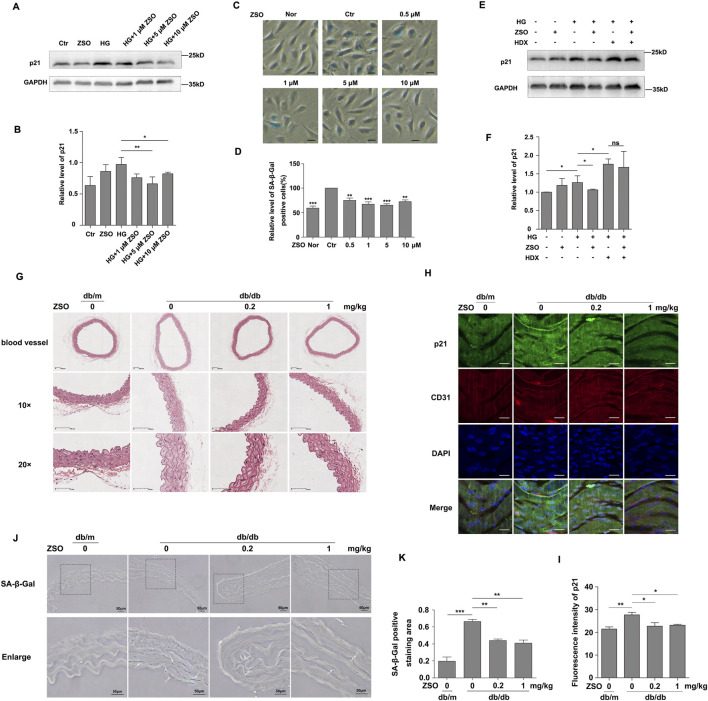
ZSO inhibits high glucose-induced senescence in vascular endothelial cells and thoracic aortic senescence in db/db mice. **(A)** Endothelial cells were treated with different concentrations of ZSO (0, 1, 5, and 10 μM) for 24 h under high glucose (30 mM) conditions. Protein level of p21 was assessed by Western blot analysis. **(B)** Quantification of the protein level of p21. **(C)** Representative SA-β-Gal staining image, showing endothelial cells treated with no treatment (Nor), high glucose (30 mM, Ctr), high glucose (30 mM) and varying concentrations of ZSO (0.5, 1, 5, 10 μM) in combination with high glucose for 24 h. Scale bar = 20 μm. **(D)** Statistical analysis of SA-β-Gal staining, representing the proportion of SA-β-Gal-positive cells relative to the total cell population. The high glucose group was considered the control (Ctr), and its statistical value was set to 1. **(E–F)** After pretreatment with 80 µM HDX for 2 h, HUVECs were incubated with ZSO (0 or 5 µM) and HDX (0 or 80 µM) in high-glucose (30 mM) conditions for 24 h. Subsequent Western blot analysis measured p21 protein levels. **(G)** Thoracic aortas were collected from mice, paraffin-embedded, and stained with hematoxylin and eosin (HE). Vascular morphology was visualized using a digital slide scanner. Representative images are shown at low magnification (full vessel view and ×10 magnification; scale bar = 100 μm) and high magnification (×20; scale bar = 50 μm). **(H)** Enface immunofluorescence staining of p21 in thoracic aorta endothelial cells. Vessels were longitudinally opened and stained for p21, CD31 (endothelial marker), and DAPI (nuclear marker), followed by confocal laser scanning microscopy. Excitation wavelengths: p21 at 488 nm, CD31 at 633 nm, and DAPI at 405 nm. Scale bar = 20 μm. **(I)** Quantitative analysis of p21 fluorescence intensity. **(J)** SA-β-Gal staining of thoracic aorta sections. Blue deposits indicate senescent cells. Scale bar = 50 μm. **(K)** Quantification of vascular senescence, expressed as the ratio of the SA-β-Gal-positive (blue-stained) area to the total vessel area. ns p > 0.05, *p < 0.05, **p < 0.01, ***p < 0.001, n = 3.

Subsequently, we investigated the effect of ZSO on HG-induced vascular endothelial cell senescence. The cell cycle-dependent kinase inhibitor p21 and senescence-associated β-galactosidase (SA-β-Gal) are well-known effectors of cell senescence (Kurz et al., 2000; Lee et al., 2006; Hernandez-Segura et al., 2018). The results revealed that high glucose treatment increased the protein levels of p21 and SA-β-Gal activity, which were effectively inhibited by treatment with 5 μM and 10 μM ZSO (Figures 3A–D). Previous studies have demonstrated that ZSO is a SO_2_ probe capable of binding to SO_2_ (Cao et al., 2016). We further explored whether its anti-senescence effects are dependent on SO_2_. To inhibit endogenous SO_2_ production in HUVECs, we treated endothelial cells with HDX (L-Aspartic acid β-hydroxamate), an inhibitor of sulfite synthetase. The experimental results demonstrated that in the absence of HDX, ZSO inhibited the high glucose-induced elevation of p21 protein levels in endothelial cells. However, following the addition of HDX, ZSO failed to suppress the increase in p21 protein levels (Figures 3E,F). These findings indicate that ZSO’s anti-senescence action relies on endogenous SO_2_.

At the end of 8 weeks of intraperitoneal ZSO treatment, the thoracic aortas of db/db mice were harvested and the vascular structure was observed by HE staining. The results showed that the blood vessels of db/m mice exhibited a compact structure, with distinct vessel wall layers and no signs of thickening or structural damage. In contrast, the blood vessels from db/db control mice displayed loose structures, thickened walls, disordered smooth muscle cell arrangement in the media, and hypertrophy of the cells. However, the blood vessels from db/db mice treated with ZSO at various doses exhibited more compact structures and milder thickening compared to the control group (Figure 3G). Enface immunofluorescence staining for p21 in the endothelial cells of the thoracic aorta was also performed. CD31, a marker of endothelial cells, was used to identify endothelial cells. The results demonstrated that p21 protein level was elevated in the db/db control group, but was significantly reduced in the ZSO-treated mice (Figures 3H,I). Additionally, frozen sections of the thoracic aorta were stained with SA-β-Gal to assess vascular senescence. The results indicated that ZSO treatment significantly inhibited high glucose-induced vascular senescence in db/db mice (Figures 3J,K). Collectively, these findings demonstrate that ZSO effectively inhibits vascular senescence in db/db mice.

### 3.4 ZSO inhibits high glucose-induced calcification in vascular endothelial cells and thoracic aortic calcification in db/db mice

**FIGURE 4:**
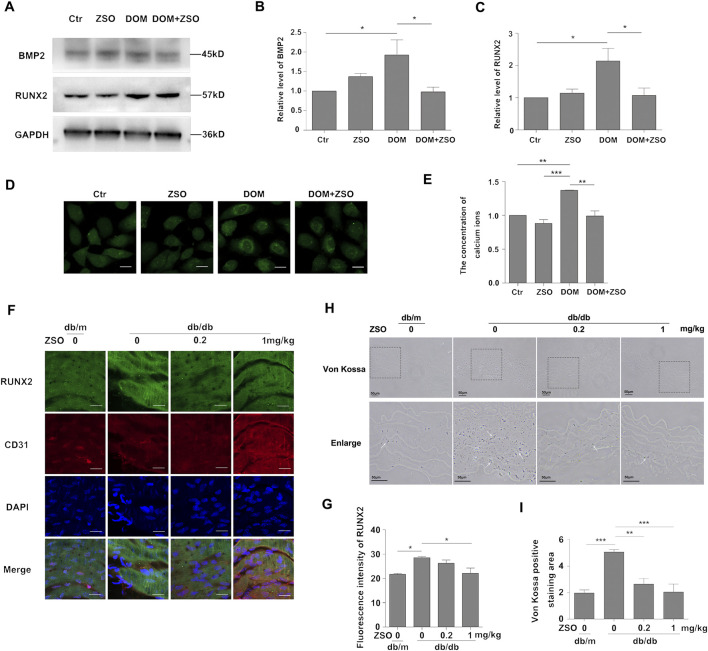
ZSO inhibits high glucose-induced calcification in vascular endothelial cells and thoracic aortic calcification in db/db mice. **(A)** Detection results of the contents of calcification-related proteins BMP2, and RUNX2. Endothelial cells were treated with no treatment, ZSO treatment alone, calcification treatment alone, calcification treatment combined with 5 μM ZSO. Western blot detection of BMP2 and RUNX2 protein levels. **(B–C)** Statistical analysis of the protein levels of BMP2 and RUNX2 respectively. **(D)** Detection of intracellular calcium ion level in endothelial cells. Intracellular calcium ion level was detected using a laser confocal microscope with excitation at 488 nm. Scale bar = 20 μm. **(E)** Quantitative analysis of intracellular calcium ion concentrations. **(F)** Enface immunofluorescence staining of mouse thoracic aorta to detect RUNX2 protein level. Confocal laser scanning microscopy was used to visualize RUNX2 (488 nm), CD31 (633 nm), and DAPI (405 nm). Scale bar = 20 μm. **(G)** Quantification of RUNX2 fluorescence intensity. **(H)** Representative image of calcium salt deposition in mouse thoracic aorta sections stained by Von Kossa, black deposits indicate positive calcium staining. Scale bar = 50 μm. **(I)** Quantitative analysis of vascular calcification based on the ratio of blue-stained area to total vessel area. *p < 0.05, **p < 0.01, ***p < 0.001, n = 3.

Cell senescence and calcification are closely interconnected, and endothelial cell senescence promotes vascular calcification. Moreover, endothelial cells can undergo endothelial-to-mesenchymal transition, differentiating into osteoblasts and chondrocytes (Yuan et al., 2021; Jiang et al., 2022). Given this, we further explored the effect of ZSO on vascular endothelial cell calcification. We established a calcification model (DOM) by treating endothelial cells with high glucose (30 mM), ascorbic acid (50 μg/mL), and β-glycerophosphate (5 mM). Intimal calcification is a hallmark of diabetes, and diabetic vascular complications, including endothelial dysfunction and vascular calcification, are often associated with bone morphogenetic proteins (BMPs) (Wu et al., 2016). Specifically, BMP2 is inflammatory mediators in endothelial cells, and its elevated activity can promote atherosclerosis and vascular calcification. RUNX2, a downstream target of BMP2, is also a key protein involved in calcification. Therefore, we evaluated the protein levels of BMP2 and RUNX2 after 4 days of calcification induction. The results showed that the levels of BMP2 and RUNX2 were significantly elevated in the calcification treatment group. However, treatment with ZSO reversed the increase in BMP2, and RUNX2 induced by calcification (Figures 4A–C). The level of intracellular calcium ions is closely related to cell calcification. The gradual increase of calcium salt accumulation is manifested as calcification. We used a calcium ion fluorescent probe to detect the calcium ion level in HUVECs, and found that the intracellular calcium ion level in the calcification treatment group was elevated, while the addition of ZSO reduced the elevated calcium ion level (Figures 4D,E). Taken together, these findings demonstrate that ZSO effectively inhibits the calcification of vascular endothelial cells induced by high glucose.

Under pathological conditions, calcium can precipitate into solid salts and deposited in tissues, a process known as ectopic or pathological calcification (Demer and Tintut, 2008; Chen et al., 2020; Dai and Khalil, 2025). Intimal calcification is recognized as a hallmark of diabetes, contributing to complications such as vascular calcification and endothelial dysfunction in advanced cases (Demer and Tintut, 2008). Previous studies have shown that db/db mice develop vascular calcification at 20 weeks (Boström et al., 2011). Therefore, we investigated the extent of vascular calcification in db/db mice and evaluated the effect of ZSO treatment. Enface immunofluorescence staining was performed to assess RUNX2 protein level in endothelial cells of the thoracic aorta. The results showed that RUNX2 was highly expressed in the db/db controls, while its expression was decreased in ZSO treated mice (Figures 4F,G). Following 8 weeks of intraperitoneal injection, calcium salt deposition in the vessel wall was assessed using Von Kossa staining, a standard method for detecting calcification. The results showed that there was significant calcium deposition in the aorta of db/db control mice, which was significantly reduced in ZSO treated db/db mice (Figures 4H,I). Taken together, these findings confirm that db/db mice develop vascular calcification and that ZSO treatment effectively inhibits this pathological process, as evidenced by reduced calcium salt deposition and RUNX2 protein level.

### 3.5 ZSO inhibits high glucose-induced vascular endothelial cell senescence and calcification through downregulating VDBP protein levels

VDBP is abundant in plasma and body fluids, where it functions as a transporter and has been recognized as a diagnostic biomarker for vascular injury, as observed in patients with STEMI and those with thrombotic plaques (Meier et al., 2006; Chishimba et al., 2010; Stakisaitis et al., 2016; Lisowska-Myjak et al., 2020). However, the relationship between VDBP and vascular endothelial cell senescence and calcification remains unknown. VDBP is closely associated with vitamin D metabolism, which plays a key role in regulating intracellular calcium level, an essential factor in cellular calcification. Based on this relationship, we hypothesized that VDBP may be involved in the process of endothelial cell calcification. This may provide novel insights for research on the relationship between endothelial cell senescence and calcification.

**FIGURE 5:**
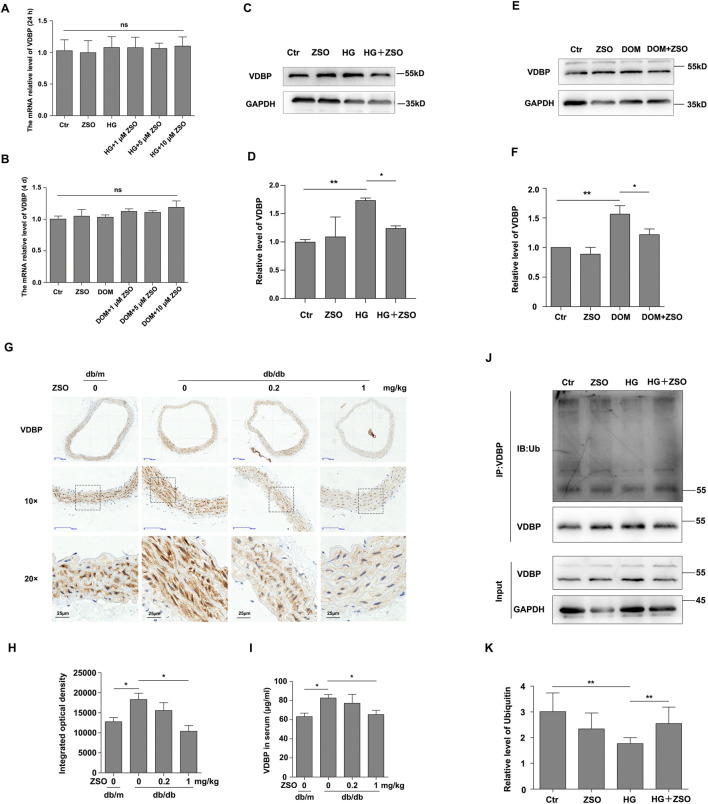
ZSO inhibits high glucose-induced vascular endothelial cell senescence and calcification through downregulating VDBP protein levels. **(A)** Endothelial cells were treated with high glucose (30 mM) and various concentrations of ZSO (1, 5, 10 μM) for 24 h. RT-qPCR was then performed to quantify VDBP mRNA level, and the results were statistically analyzed. **(B)** Endothelial cells were treated with DOM and various concentrations of ZSO (1, 5, 10 μM) for 4 days. RT-qPCR was then performed to quantify VDBP mRNA level, and the results were statistically analyzed. **(C)** Endothelial cells were treated with high glucose (30 mM) and ZSO (5 μM) for 24 h, followed by Western blot analysis of VDBP protein level. **(D)** Statistical analysis of VDBP protein level under senescence conditions. **(E)** Endothelial cells were treated with 5 μM ZSO under calcification-inducing conditions for 4 days, followed by Western blot analysis of VDBP protein level. **(F)** Quantitative analysis of VDBP protein level under calcification conditions. **(G)** Immunohistochemical staining of VDBP in mouse thoracic aorta sections, with brown coloration indicating positive immunoreactivity. **(H)** Quantitative analysis of VDBP-positive staining intensity in vascular tissues, with corresponding statistical graphs. **(I)** Measurement of VDBP protein level in mouse serum using an ELISA kit, followed by statistical analysis. **(J)** Endothelial cells were treated with high glucose (30 mM) and 5 μM ZSO for 24 h, and the ubiquitination level of VDBP was assessed. **(K)** Quantification of VDBP ubiquitination level. ns p > 0.05, *p < 0.05, **p < 0.01, n = 3.

Preliminary studies have demonstrated that ZSO can bind to VDBP and inhibit vascular senescence and calcification. This raises the question of whether VDBP plays a crucial role in mediating ZSO’s regulatory effects on vascular endothelial cell senescence and calcification. To further investigate the relationship between VDBP and vascular endothelial cell senescence/calcification, we measured VDBP mRNA and protein levels in high glucose-induced senescent and calcified endothelial cells. The results showed no significant difference in VDBP mRNA levels under high glucose conditions, whereas VDBP protein levels were elevated. ZSO treatment effectively suppressed this high glucose-induced increase in VDBP protein (Figures 5A–F). Additionally, elevated VDBP protein levels were detected in the thoracic aortas and serum of db/db mice, and ZSO administration inhibited this elevation (Figures 5G–I). To elucidate the mechanism by which ZSO downregulates VDBP protein levels, we examined the intracellular pathway and discovered that during high glucose-induced endothelial cell senescence, ZSO promotes ubiquitination to reduce VDBP protein levels (Figures 5J,K). Collectively, these findings indicate that under high glucose conditions, decreased ubiquitination of VDBP leads to its protein accumulation during vascular endothelial cell senescence. After ZSO treatment, ZSO binds to VDBP, enhancing its ubiquitination-mediated degradation, thereby reducing VDBP protein levels and inhibiting high glucose-induced vascular endothelial cell senescence and calcification.

## 4 Discussion

**FIGURE 6:**
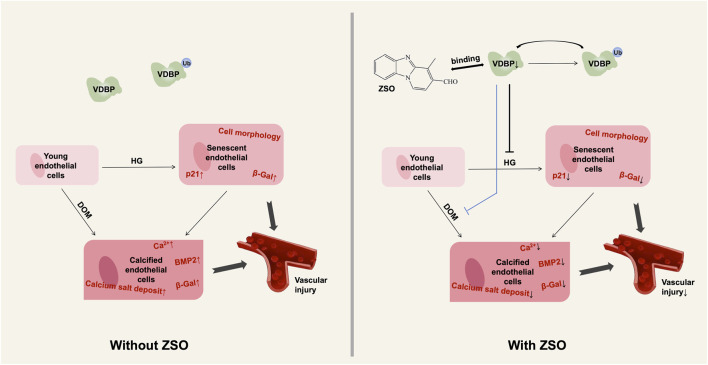
Schematic diagram of the mechanism by which ZSO inhibits vascular endothelial cell senescence and calcification. ZSO binds to the VDBP, promoting its ubiquitin-mediated degradation. This process suppresses high glucose-induced senescence in vascular endothelial cells and further inhibits their calcification, thereby reducing vascular damage caused by external stimuli.

Endothelial cell senescence is crucial for vascular aging and the development of diabetes-related vascular complications. VDBP, a protein abundant in plasma and body fluids, is involved in the transport of multiple ligands and regulates various physiological and pathological processes (Lisowska-Myjak et al., 2020). Elevated levels of VDBP have been linked to systemic inflammation and cardiovascular injury (Kalousova et al., 2015; Stakisaitis et al., 2016; Lisowska-Myjak et al., 2020). However, its role in endothelial senescence remains unexplored, and no known small molecules capable of binding to VDBP to modulate its protein levels have been reported to date. In this study, we identified a sulfur dioxide probe ZSO capable of binding to VDBP protein. Both *in vitro* and *in vivo* experiments demonstrated that ZSO effectively inhibits high glucose-induced endothelial cell senescence and calcification. During vascular endothelial cell senescence and calcification, VDBP protein levels were upregulated, while ZSO facilitated ubiquitination-mediated degradation of VDBP, thereby downregulating VDBP protein expression and suppressing high glucose-induced endothelial cell senescence and calcification (Figure 6). This work provides the first evidence establishing the association between VDBP and vascular endothelial cell senescence, while elucidating the molecular mechanism by which the SO_2_ probe ZSO regulates VDBP to inhibit hyperglycemia-induced endothelial aging. These findings offer novel scientific rationale and identify promising therapeutic targets for preventing diabetic vascular complications. Furthermore, VDBP emerges as a potential diagnostic biomarker for diabetic vascular complications, introducing innovative perspectives for clinical detection methodologies.

Research indicates that alterations in VDBP levels may be influenced by various diseases and could serve as indicators of disease progression. VDBP exhibits distinct expression patterns during chronic and acute phases of different conditions (Lisowska-Myjak et al., 2020). For instance, elevated VDBP expression has been observed in serum from patients with fresh thrombotic plaques and STEMI, suggesting its potential as a biomarker for vascular injury (Stakisaitis et al., 2016; Lisowska-Myjak et al., 2020). These findings have important prognostic and diagnostic implications. In this study, we further observed that VDBP is upregulated during high glucose-induced vascular senescence and calcification. This finding highlights its role in vascular stress responses and extends our appreciation of its functional diversity.

Currently, the role of VDBP in senescence remains unidentified. In this study, we demonstrated in both *in vitro* and *in vivo* models that ZSO effectively antagonizes high glucose-induced vascular endothelial cell senescence, evidenced by suppressed p21 protein expression and β-galactosidase activity. Notably, this anti-senescent effect relies on endogenous SO_2_ synthesis pathways, highlighting the critical role of endogenous SO_2_ in combating aging (Cao et al., 2016). As an SO_2_ probe, ZSO binds endogenous SO_2_, thereby enabling us to functionally connect SO_2_ signaling to the regulation of VDBP. Furthermore, ZSO treatment did not affect VDBP mRNA levels but promoted its ubiquitination-mediated degradation, thereby reducing VDBP protein stability. We hypothesize that this SO_2_-dependent ubiquitination of VDBP may be mediated by a post-translational modification known as sulfenylation (S-sulfenylation, the formation of -SOH on cysteine residues). The oxidative modification and conformational changes of VDBP might enhance its affinity for E3 ubiquitin ligases, initiating the process of ubiquitination-mediated proteasomal degradation. However, the mechanistic relationship between SO_2_ signaling, VDBP sulfenylation, and ubiquitination requires further investigation.

VDBP may influence vascular calcification by regulating vitamin D bioavailability and calcium-phosphate homeostasis (Martínez-Sena et al., 2020), however, no direct evidence currently links VDBP to vascular calcification. Our *in vitro* and *in vivo* experiments demonstrated that ZSO effectively inhibited calcification, thereby preventing the progression from endothelial dysfunction to vascular calcification. Importantly, VDBP was significantly elevated in senescent endothelial cells and diabetic mouse vasculature, suggesting its role as a key mediator linking senescence and calcification processes. This finding positions VDBP as a novel therapeutic target in vascular biology and diabetic vasculopathy. Moreover, the observed elevation of serum VDBP levels in diabetic mice and its reduction following ZSO intervention suggest the potential of serum VDBP as a biomarker for monitoring diabetic vascular complications.

Collectively, these discoveries reveal a novel mechanism in regulating endothelial senescence and vascular calcification, providing fresh perspectives for understanding the molecular basis of diabetic vascular lesions. However, the precise mechanisms of molecular crosstalk by which ZSO modulates VDBP signaling requires further research to clarify.

In conclusion, this study identifies VDBP as a key mediator in high glucose-induced endothelial cell senescence and proposes a novel molecular mechanism through which ZSO regulates VDBP to inhibit high glucose-induced vascular aging. These findings offer new insights into the management of diabetes-related vascular complications and highlight the potential of VDBP as a clinical biomarker for diabetic vascular injury.

## Data Availability

The original contributions presented in the study are included in the article/Supplementary Material, further inquiries can be directed to the corresponding authors.
