# Supplementary material for: A new SO2 probe ZSO targeting VDBP inhibits high glucose induced endothelial cell senescence and calcification
Source: Front Physiol. 2026 Jan 5;16:1719853. doi: 10.3389/fphys.2025.1719853 (PMC12812760; doi:10.3389/fphys.2025.1719853)

# Figure 3A original data

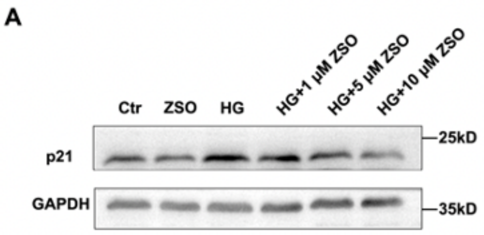


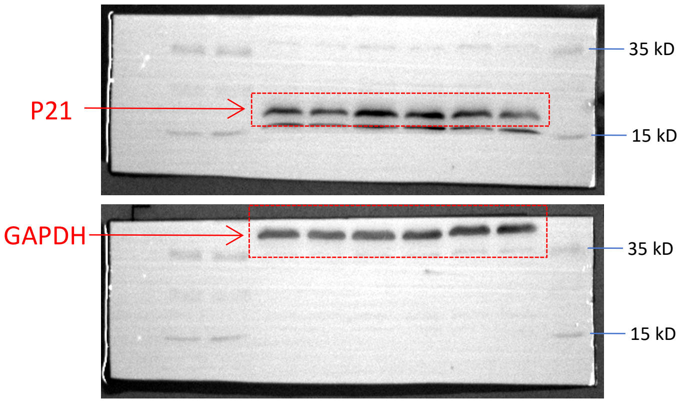


Protein marker

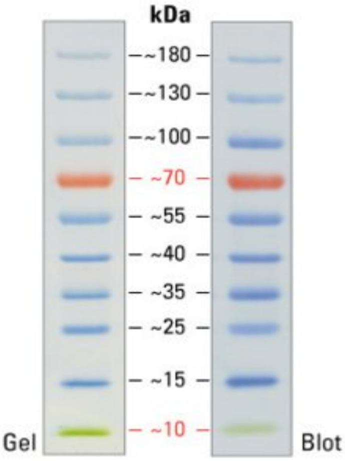

## Figure 3E original data

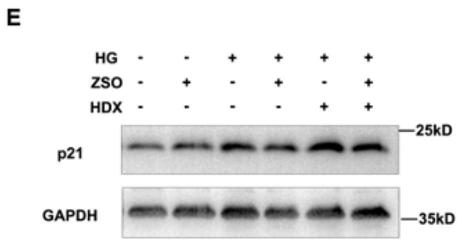


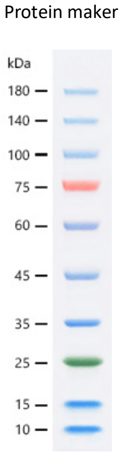


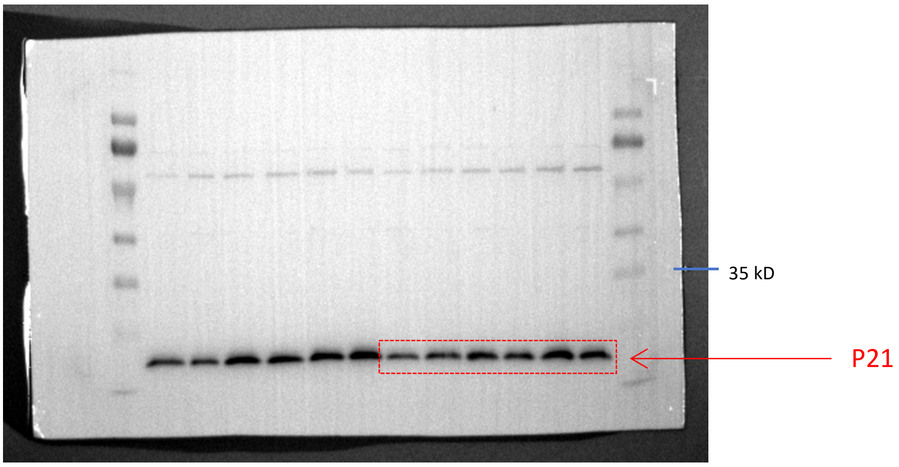


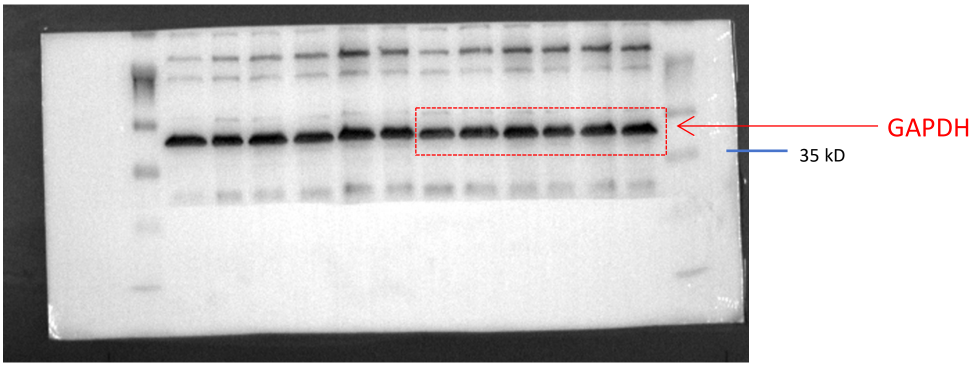

# Figure 4A original data

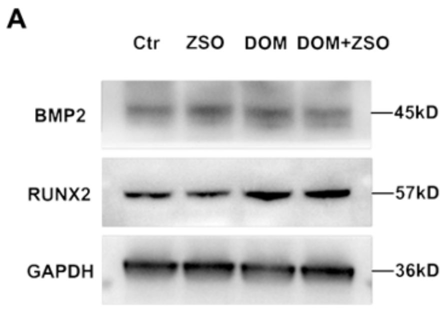


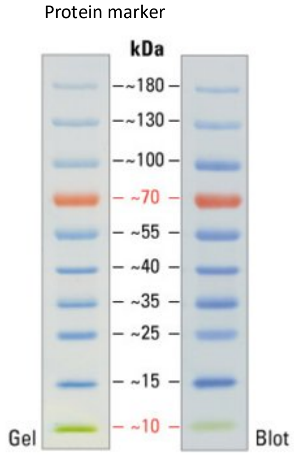


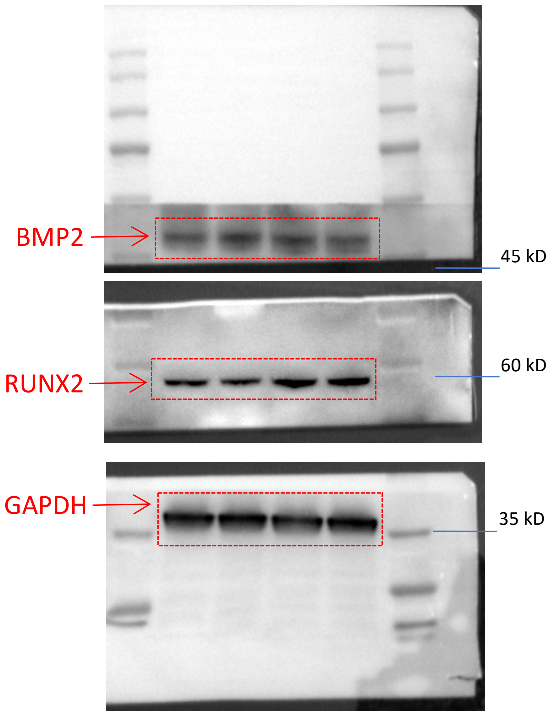

## Figure 5C original data

C

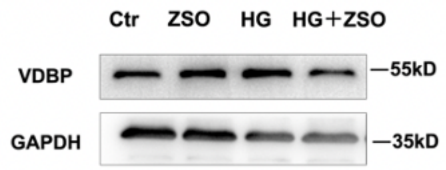


Protein marker

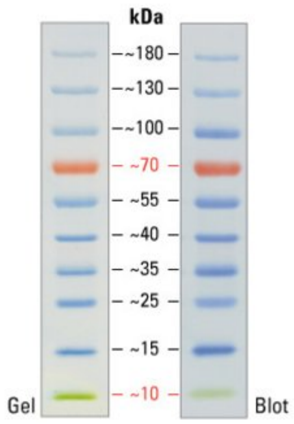


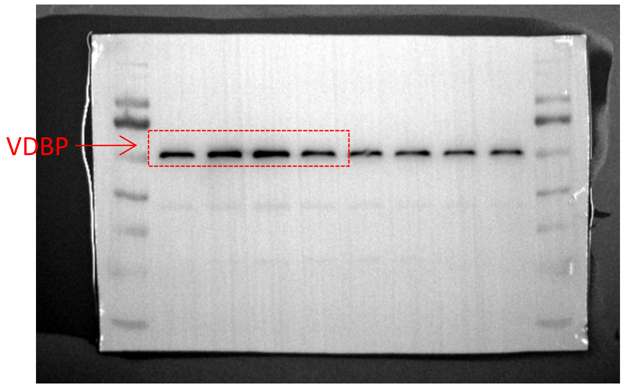


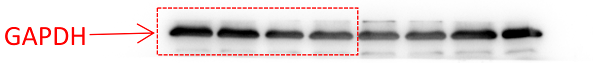


GAPDH- with marker

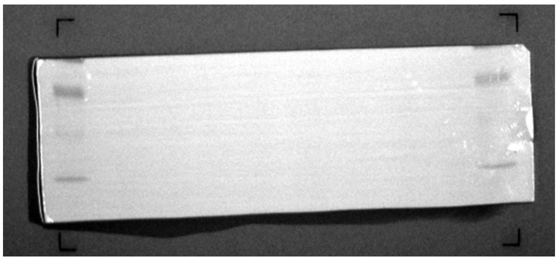

# Figure 5E original data

**E**

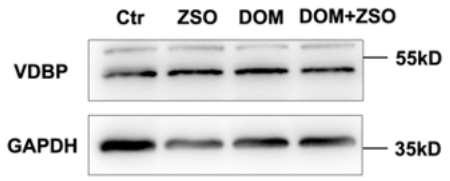


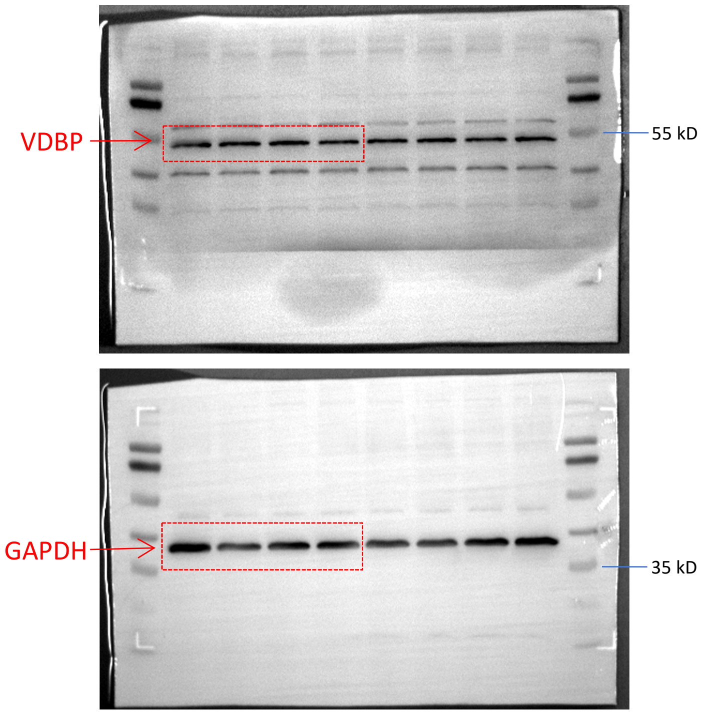


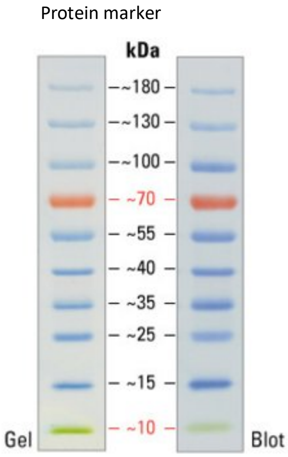

# Figure 5J original data

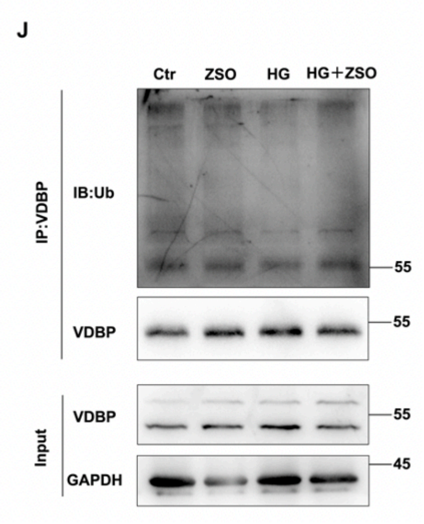


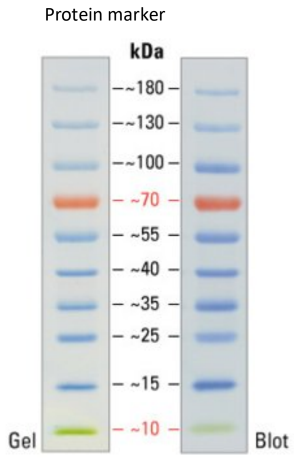


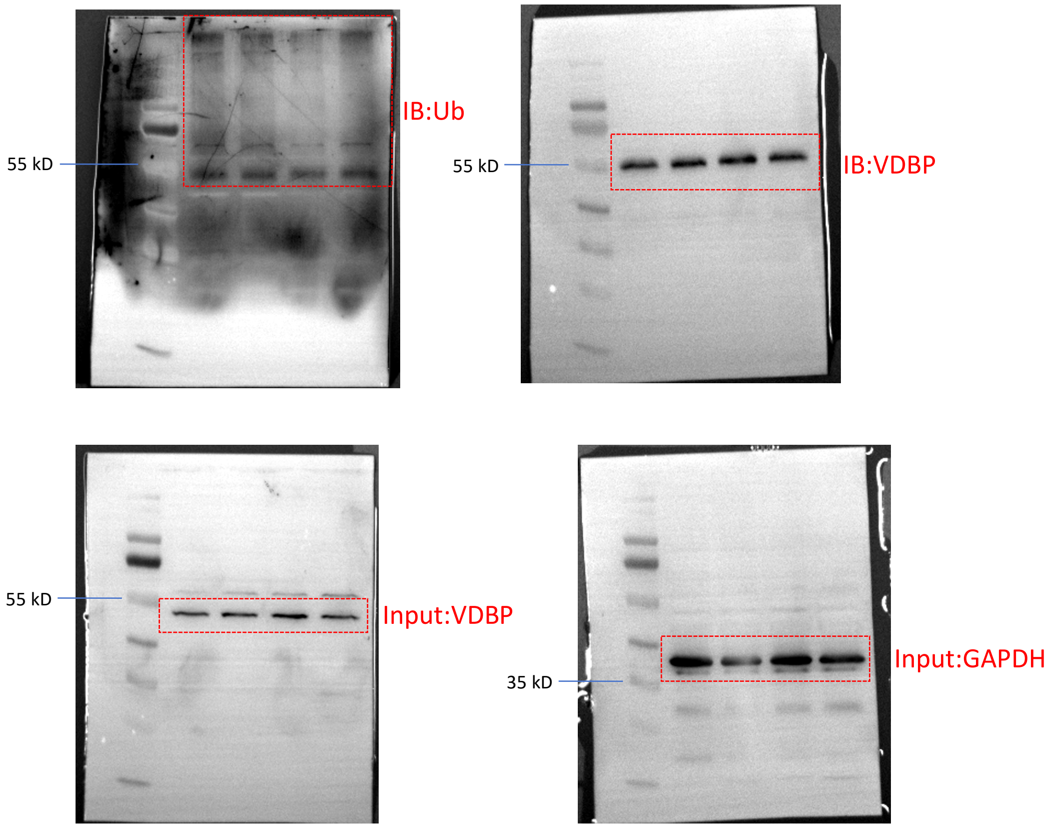

Supplement: Supplementary file 1 [file DataSheet1.pdf]
